# Author Correction: Unique and universal dew-repellency of nanocones

**DOI:** 10.1038/s41467-021-24355-7

**Published:** 2021-06-28

**Authors:** Pierre Lecointre, Sophia Laney, Martyna Michalska, Tao Li, Alexandre Tanguy, Ioannis Papakonstantinou, David Quéré

**Affiliations:** 1 Physique et Mécanique des Milieux Hétérogènes, UMR 7636 du CNRS, ESPCI, PSL Research University, Paris, France; 2 LadHyX, UMR 7646 du CNRS, École Polytechnique, Institut Polytechnique de Paris, Palaiseau, France; 3 Photonic Innovations Lab, Department of Electronic and Electrical Engineering, University College London, London, UK; 4 Laboratoire de Mécanique des Solides, UMR 7649 du CNRS, École Polytechnique, Institut Polytechnique de Paris, Palaiseau, France

**Keywords:** Mechanical engineering, Mechanical properties

Correction to: *Nature Communications* 10.1038/s41467-021-23708-6, published online 8 June 2021.

The original version of this Article contained an error in the Additional Information section.‘D.Qéré’ should have been written as ‘D.Q.’ This has now been corrected in both the PDF and HTML versions of the Article.

