# Supplementary material for: Unique and universal dew-repellency of nanocones
Source: Nat Commun. 2021 Jun 8;12:3458. doi: 10.1038/s41467-021-23708-6 (PMC8187394; doi:10.1038/s41467-021-23708-6)
Supplement: Supplementary file 1 — Supplementary Information [file 41467_2021_23708_MOESM1_ESM.pdf]

Unique and universal dew-repellency of nanocones

Supplementary information

Supplementary Figures

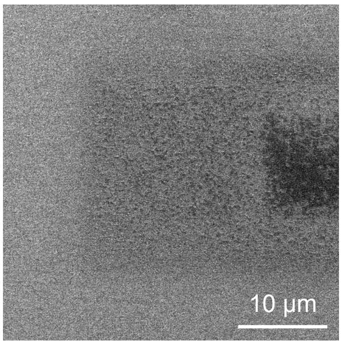

Supplementary Figure 1. Effects of high energy e-beam on our samples. Long exposure to e-beam degrades the surface, as revealed here by looking at water condensation on sample H1. The dark square area was exposed for few minutes to a beam of 15 keV. Dark stains show filmwise water condensation, in contrast with the expected behavior on a superhydrophobic surface where droplets exhibit high contact angles. This observation then strengthens a possible silane coating deterioration.

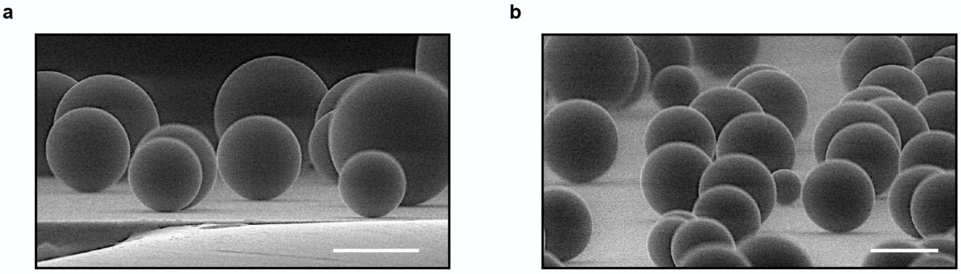

Supplementary Figure 2. ESEM images of water condensing on samples E2' (*h* = 220 nm) **(a)** and E3 (*h* = 284 nm) **(b)**, two other materials of the E family. As for material E4 in the accompanying paper, drops are all quasi-spherical at the scale of 10 µm, a manifestation of their Cassie state. Scale bars indicate 10 µm.

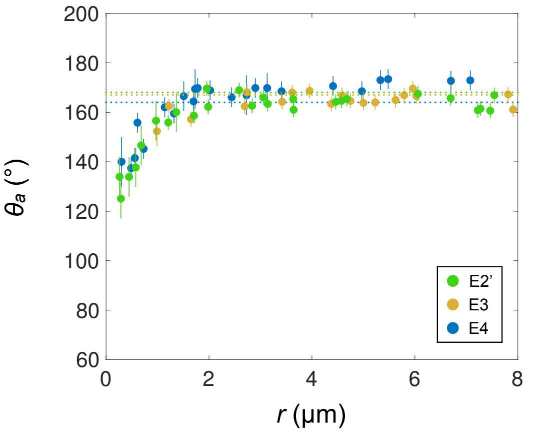

Supplementary Figure 3. Contact angle of microdroplets condensing on nanocones. Advancing contact angle θ_a measured by ESEM imaging as a function of the droplet radius r for materials E2' (green dots), E3 (yellow dots) and E4 (blue dots). In all cases, the contact angle increases and saturates with r. For all three samples, data collapse on the same curve at small radii ($r < 2$ µm). At larger radii, data approach the contact angles of millimetric water drops drawn with dotted lines: $\theta_a = 168° \pm 3°$, $167° \pm 3°$ and $164° \pm 3°$ for E2', E3 and E4, respectively.

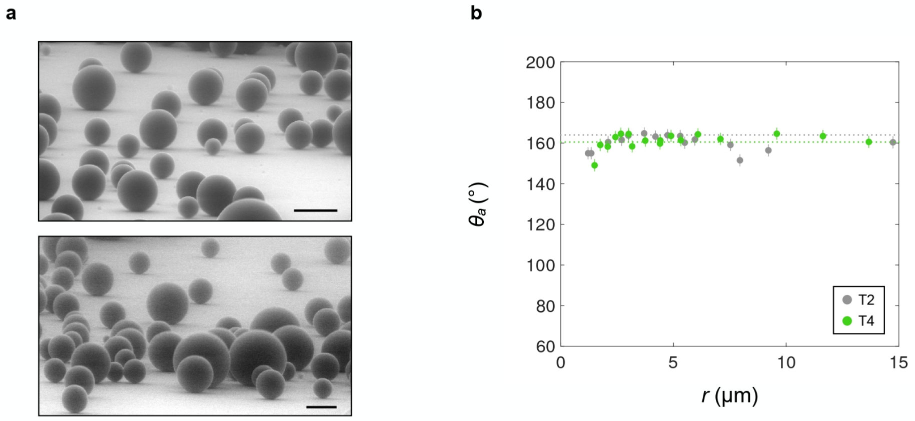

Supplementary Figure 4. a. ESEM images of water condensation on T2 (top image, top diameter l = 42 nm) and T4 (bottom image, l = 60 nm). All droplets exhibit a quasi-spherical shape and their contact angles approach ~160°. The low contact area with the solid suggests a Cassie state. Both scale bars indicate 10 µm. **b.** Advancing contact angle θ_a as a function of the drop radius r for samples T2 and T4. θ_a increases towards the value measured on millimetric drops (dashed lines): $\theta_a = 164 \pm 1°$ and $160.5 \pm 1°$ for T2 and T4, respectively.

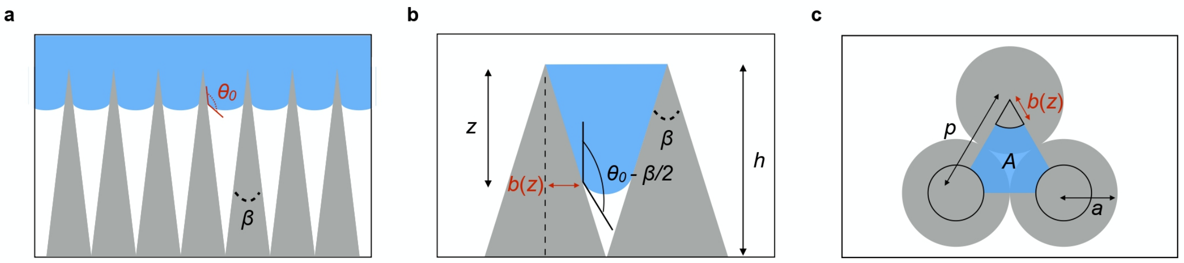

Supplementary Figure 5. a. Cassie state for a drop resting on a dense array of cones. **b.** Side view of liquid inside the cones. The contact line sinks at a depth z and it makes an angle $\theta_0 - \beta/2$ with the vertical, denoting β as the cone angle and θ_0 as the Young contact angle. The radius of the cone at the contact line is denoted as $b(z)$. **c.** Top view of an elementary triangular cell delimited by three cones. The liquid occupies an area A.

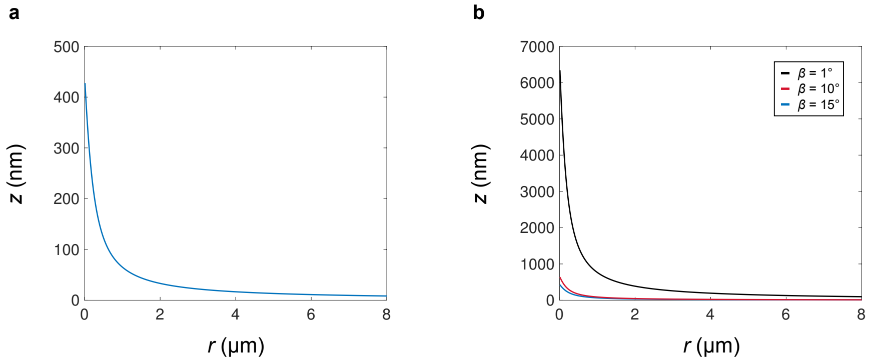

Supplementary Figure 6. a. Sinking depth z for a drop with radius r in a Cassie state on an array of hydrophobic cones. The solid line is eq. (1) plotted for sample E4 (p = 110 nm and h = 420 nm). The depth z decreases hyperbolically with the drop radius r. **b.** Sinking depth z for a drop with radius r for three values of cone angle β and using eq. (1): β = 1° (black line), 10° (red line) and 15° (blue line). The pitch is p = 110 nm and h = 6.3 μm, 630 nm and 420 nm with respective β = 1°, 10° and 15°. Sharper cones induce water penetration.

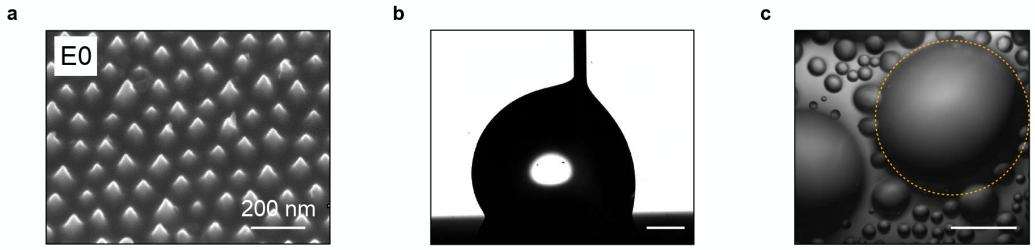

Supplementary Figure 7. a. SEM images of the nanocones E0 with pitch p = 110 nm and reduced height h = 101 nm. They are arranged on a hexagonal array and coated by a hydrophobic layer. **b.** A water droplet on E0 simply exhibits a slightly obtuse angle, in stark contrast with the other samples of family E. Water impregnates the texture and falls in the Wenzel state, owing to the large apex angle, $\beta = 57° \pm 2°$, smaller than the critical angle β_c = 60° for cavity penetration. The scale bar indicates 500 μm. **c.** Breath figure on sample E0 under optical microscope after 25 min. The surface is largely covered by water and droplets are deformed (highlighted by the dotted circle). No jump is observed during 30 min. The scale bar indicates 100 μm.

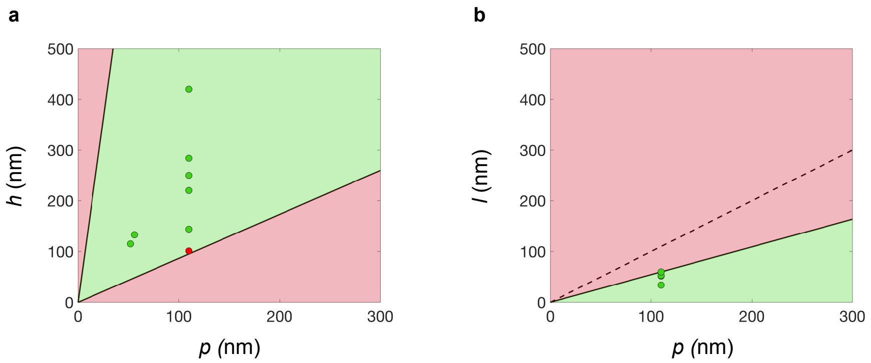

Supplementary Figure 8. a. Phase diagram of antifogging on sharp nanocones. The two control parameters are the cone height h and the pitch p. The two limits are $h = p/2\ \tan(\beta/2)$, where β is 4° and 60°. Observations (green and red symbols for jumping and non-jumping, respectively) with the eight samples (extruded and homothetic families) are found to all agree with the model. **b.** Phase diagram of antifogging on truncated nanocones. The two control parameters are the cone top diameter l and the pitch p. The dotted and solid lines respectively show the limit $l = p$ and the equation $p = (\pi/2\sqrt{3}\varphi_s)^{1/2}\ l$, where φ_s is the critical solid fraction (see eq. S2) for which the drop contact angle is equal to 150°. The latter line delimitates the frontier between antifogging (green) or not (red). Observations made at fixed p with four truncated cones (T family) all agree with the model.

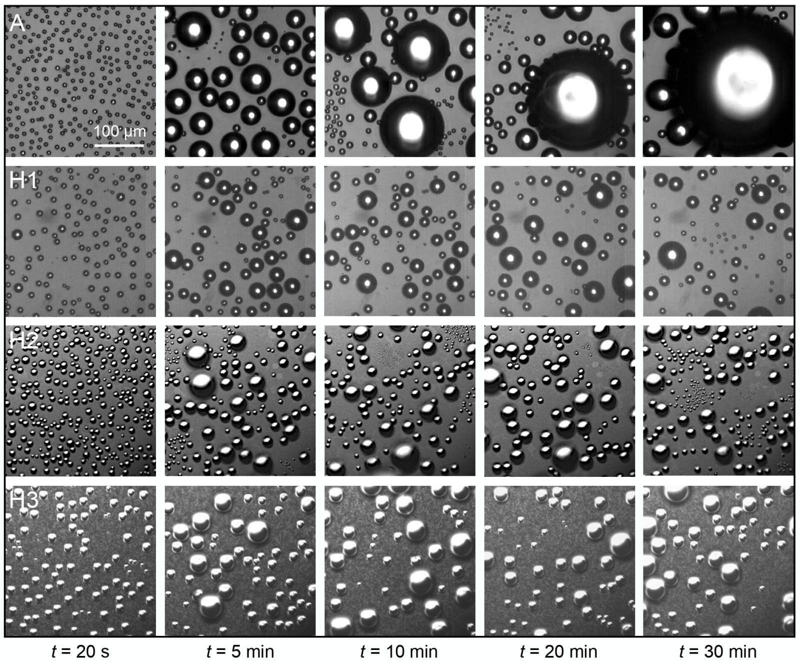

Supplementary Figure 9. Breath figures on pillars A and on the homothetic family H (samples H1-H2-H3) under an optical microscope after 20 s, 5 min, 10 min, 20 min and 30 min. Room temperature is $T = 21 \pm 1$°C, relative humidity $RH = 52 \pm 2$% and sample temperature is $T_s = 4 \pm 1$°C, which corresponds to a supersaturation $S = 1.6 \pm 0.2$. Differences between pillars and sharp cones become apparent after 5 min: sample A is covered by larger and larger droplets while, for all structures H, growing droplets coalescing with their neighbors can jump off the surface, which results in similar breath figures.

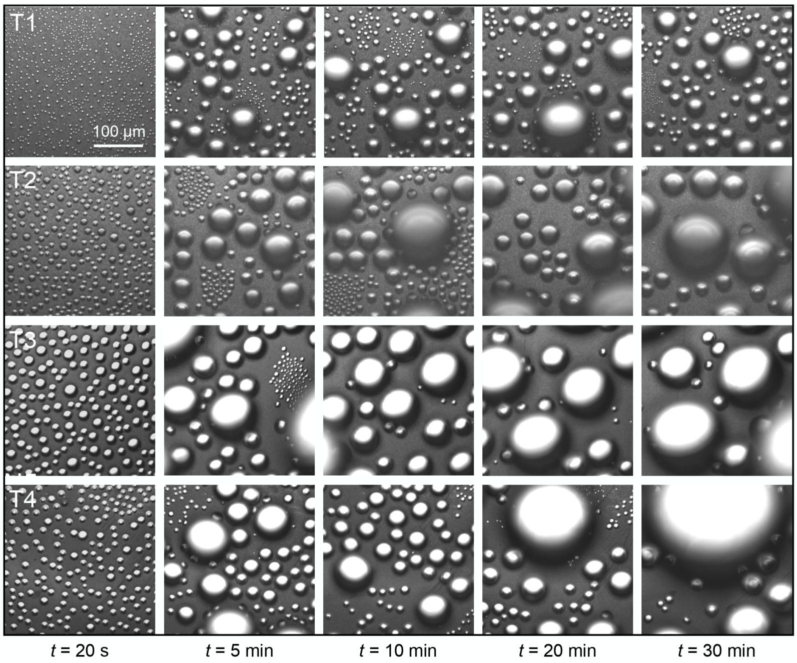

Supplementary Figure 10. Breath figures on the truncated family T (samples T1-T2-T3-T4) under optical microscope after 20 s, 5 min, 10 min, 20 min and 30 min. Room temperature is $T = 21 \pm 1$°C, relative humidity $RH = 52 \pm 2$% and sample temperature is $T_s = 4 \pm 1$°C, which corresponds to $S = 1.6 \pm 0.2$. We observe a hierarchy between the samples: the more truncated, the more water-covered the sample; T1 appears to be the most antifogging among the four samples.

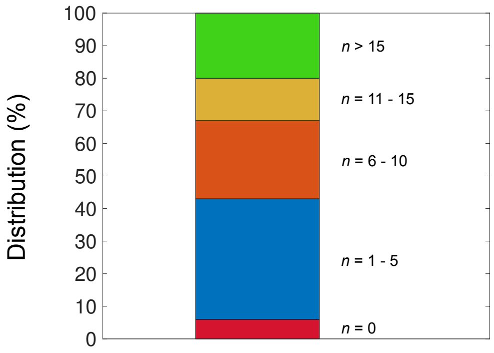

Supplementary Figure 11. Histogram of renucleation on material H1. For 150 nuclei initially present, we measure the number n of nuclei re-appearing at the same location after the drop jumped, while the sample temperature is kept constant at $T_s = 4 \pm 1$°C. The distribution of re-nucleation is shown as a function of n.

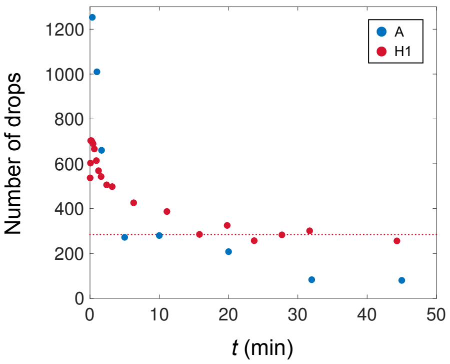

Supplementary Figure 12. Absolute number of drops on sample H1 (red dots) and A (blue dots) as a function of time. For H1, the number first rises, then decreases until plateauing after 15 min; in contrast, it keeps decreasing on A. The red line marks the plateau observed on nanocones.

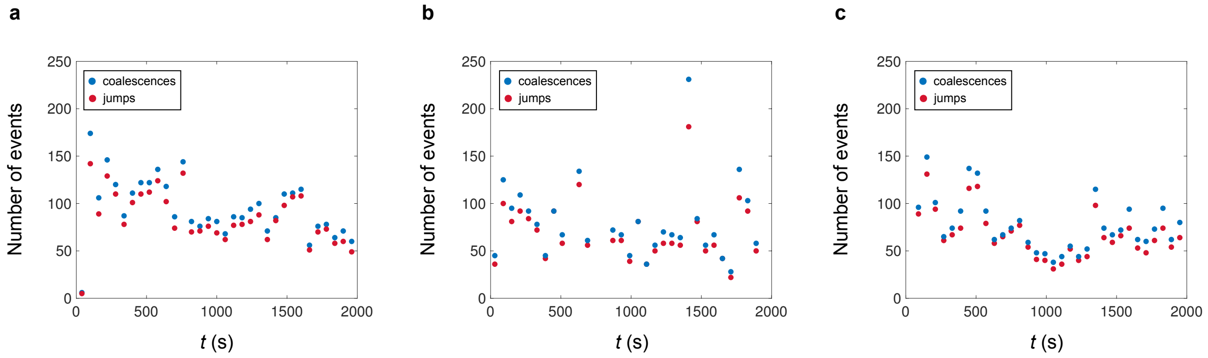

Supplementary Figure 13. Absolute number of coalescences and jumps as a function of time t for materials decorated with nanocones. Each data point is obtained after measuring these numbers over one minute. For all samples, we successively observe an increase, a decrease and a stationary regime after ~500 s. Importantly, the two events strongly correlate, regardless of the amplitude of the observed fluctuations. **a.** Data on sample H1. **b.** Data on sample H3/E2. **c.** Data on sample E4.

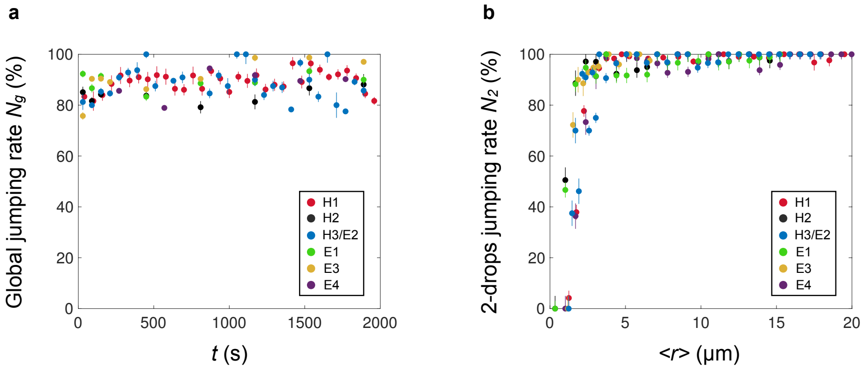

Supplementary Figure 14. a. Global jumping rate N_g as a function of time t for all samples H and E. Each data point is obtained by averaging the proportion of jumps over one minute after considering all types of coalescences. N_g is roughly constant over time and it is ~90% for the three materials. **b.** Jumping rate N_2 for symmetric binary coalescences, where the drop radii do not differ by more than 20%. N_2 is plotted as a function of the average drop size $\langle r \rangle$ for families H and E. In all cases, the jumping rate plateaus at 99 ± 1% above a critical radius $r_c \approx 1.5 \pm 0.4$ μm.

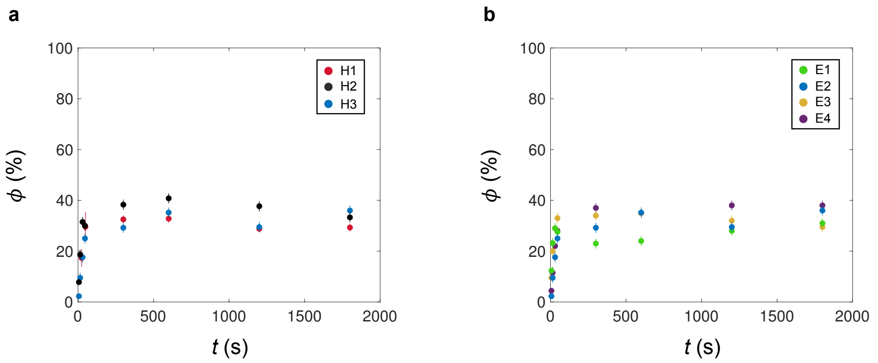

Supplementary Figure 15. a. Area fraction ϕ covered by water as a function of time t for samples H1-H3. **b.** Area fraction ϕ covered by water as a function of time t for samples E1-E4. In both cases, data are similar and no hierarchy can be established between materials.

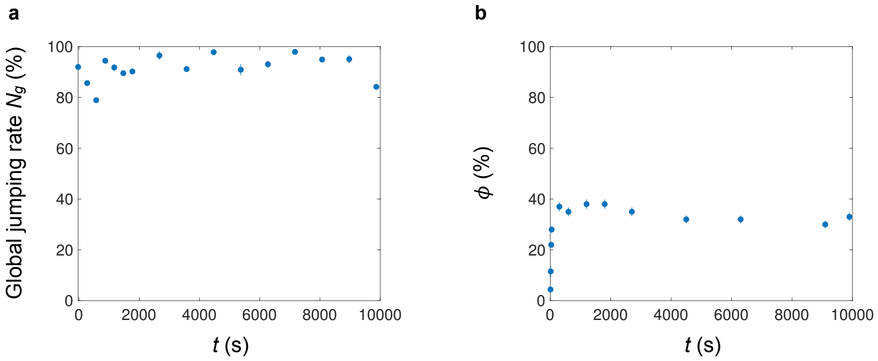

Supplementary Figure 16. a. Global jumping rate N_g as a function of time t for sample E4, over a longer time period. The percentage of jumps remains quasi-constant over nearly 3 hours. **b.** Area fraction ϕ covered by liquid as a function of time t for sample E4. It stabilizes at a value of around 33% for the whole duration of the experiment.

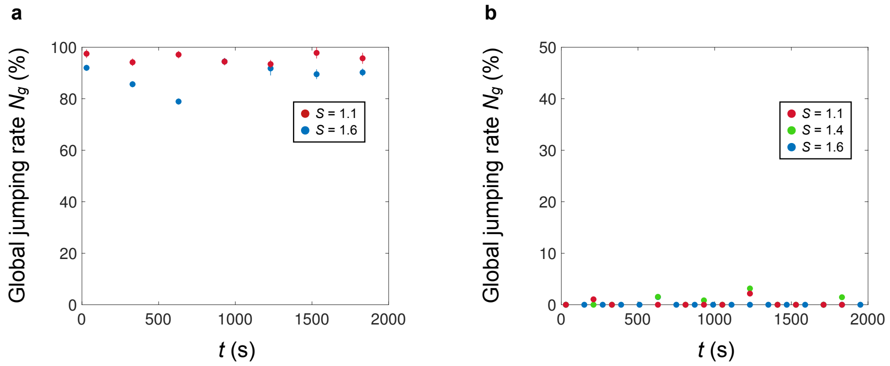

Supplementary Figure 17. a. Global jumping rate N_g as a function of time t for sample E4 for two values of supersaturations S: S = 1.1 (red dots) and S = 1.6 (blue dots). Each point is obtained after measuring N_g over one minute. **b.** Global jumping rate N_g as a function of time t for sample A for three values of the supersaturations S: S = 1.1 (red dots), S = 1.4 (green dots) and S = 1.6 (blue dots). Whatever S, the percentage of jumps remains negligible.

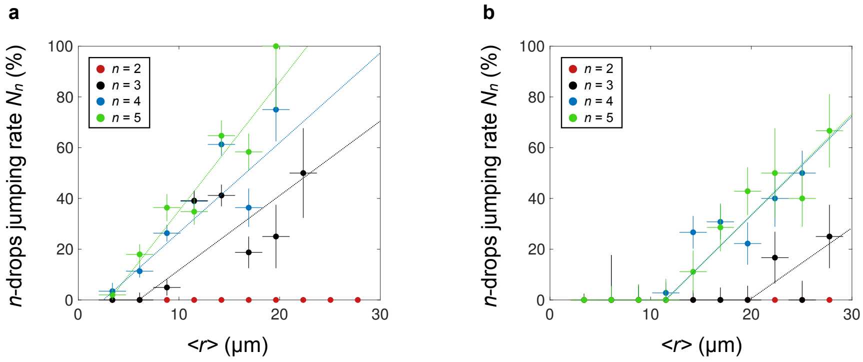

Supplementary Figure 18. a. Jumping rate N_n of droplets on T1 as a function of the mean radius <r> of merging drops, after distinguishing among all coalescences the ones that imply n = 2, 3, 4 or 5 droplets. Dotted lines are a guide for the eyes. **b.** Jumping rate N_n of droplets on T4 as a function of the mean radius <r> of merging drops. N_n increases with both n and <r>. Compared to T1, N_n is shifted towards larger droplet radii. Dotted lines are a guide for the eyes.

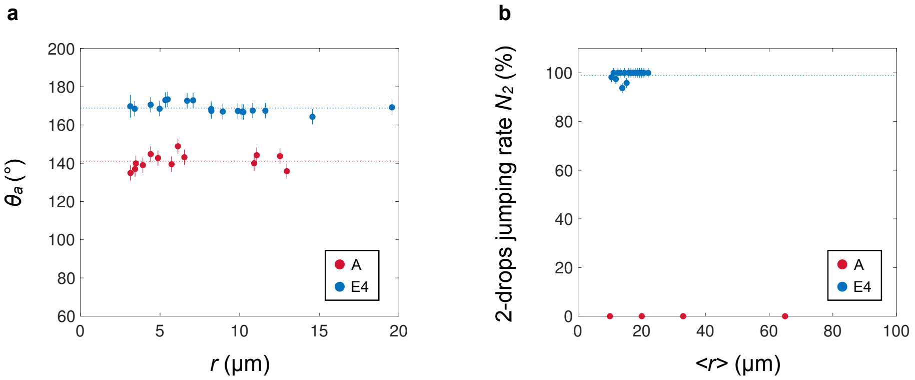

Supplementary Figure 19. a. Advancing contact angle θ_a measured by ESEM as a function of the droplet radius r for samples A (nanopillars, red dots) and E4 (nanocones, blue dots). In both cases, the contact angle plateaus with r (dotted lines), at θ_a = 141° ± 6° for A and 169° ± 6° for E4. **b.** Jumping rate N_2 for symmetric binary coalescences as a function of the average drop size <r> for nanopillars A and nanocones E4. The radius of jumping droplets does not exceed 25 μm on sample E4 because of the high antifogging efficiency. In contrast, it can reach 65 μm (or more) on A with no jumping, independently of <r>.

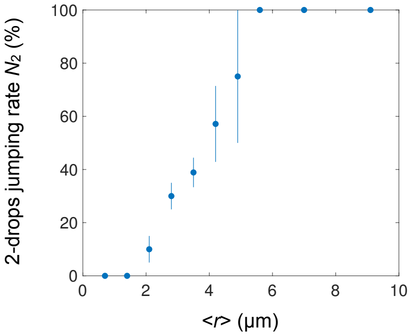

Supplementary Figure 20. Jumping rate N_2 for symmetric binary coalescences as a function of the average drop size <r> on nanoneedles with h = 600 nm, p =300 nm and an aspect ratio of 6. Contrasting with short nanopillars, drops can jump if their radius is larger than typically 5 μm. Data extracted from ref. 12.

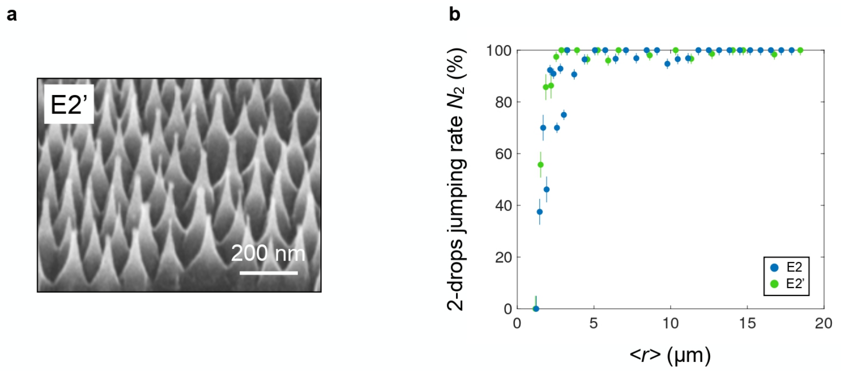

Supplementary Figure 21. a. Scanning electron micrographs of the nanocones E2’ ($h = 220$ nm and $p = 110$ nm) disposed on a dense hexagonal array and covered by a hydrophobic layer. In contrast with its counterparts from the E family, cones are convex with a varying cone angle. **b.** Jumping rate N_2 for binary, symmetric coalescences, as a function of droplet size for samples E2 and E2’. All data collapse on a single curve with a critical radius of jump $r_c \approx 1.6 \pm 0.3$ µm and 1.5 ± 0.3 µm, for E2 and E2’ respectively, and a plateau value of 99 ± 1%. This curve is very similar to those obtained in figures 4c and 4d.

Supplementary Tables

	p **(nm)**	***h*** **(nm)**	***l*** **(nm)**	***β*** **(°)**	$\boldsymbol{\theta_a}$ **(°)**	$\boldsymbol{\theta_r}$ **(°)**
**A**	52	88	30	N/A	167	140
**H1**	52	115	N/A	25	167	157
**H2**	56	133	N/A	24	166	154
**H3/E2**	110	250	N/A	23.5	169	159
**E0**	110	101	N/A	57	132	23
**E1**	110	144	N/A	38		
**E2’**	110	220	N/A	28	168	153
**E3**	110	284	N/A	21	167	161
**E4**	110	420	N/A	15	164	153
**T1**	110	258	34	17		
**T2**	110	162	42	24	164	138
**T3**	110	107	53	30	163.5	130
**T4**	110	117	60	24	160.5	131

Supplementary Table 1. Characteristics of the nanotextured materials. Sample A comprises nanopillars, family H consists of homothetic cones, extruded cones E are more and more elongated and cones T are truncated. Families H and E have a common member called either H3 or E2. The cones (of angle β) are spaced with a pitch p and have a height h and possibly a top diameter l. In each family, the samples are ranked from lowest to largest characteristic size. Contact angles are measured with millimeter size water drops, and we provide both the advancing and receding values of these angles, measured with an uncertainty of ± 2°.

r (μm)	[3.4 - 6.1[		[6.1 - 8.8[		[8.8 - 11.5[		[11.5 - 14.2[		[14.2 - 16.9[		[16.9 - 19.6[		[19.6 - 22.3[		[22.3 - 25[	
	N jumps	N coal	N jumps	N coal	N jumps	N coal	N jumps	N coal	N jumps	N coal	N jumps	N coal	N jumps	N coal	N jumps	N coal
$n = 3$	0	103	0	74	3	61	18	46	14	34	3	16	1	4	1	2
	0 %		0 %		5 %		39 %		41 %		19 %		25 %		50 %	
$n = 4$	2	58	11	97	15	57	14	36	19	31	4	11	3	4	0	0
	3 %		11 %		26 %		39 %		61 %		36 %		75 %		N/A	
$n = 5$	2	40	7	39	8	22	8	23	11	17	7	12	1	1	0	0
	2 %		18 %		36 %		35 %		65 %		58 %		100 %		N/A	

Supplementary Table 2. Characteristics of the jumping rates N_n on sample T1. For each range of drop radii, we provide the number of coalescences and jumps for $n = 3$, 4 and 5, denoting n as the number of drops involved in the coalescence.

r (μm)	[3.4 - 6.1[		[6.1 - 8.8[		[8.8 - 11.5[		[11.5 - 14.2[		[14.2 - 16.9[		[16.9 - 19.6[		[19.6 - 22.3[		[22.3 - 25[		[25 - 27.7[		[27.7 - 30.4[	
	N jumps	N coal	N jumps	N coal	N jumps	N coal	N jumps	N coal	N jumps	N coal	N jumps	N coal	N jumps	N coal	N jumps	N coal	N jumps	N coal	N jumps	N coal
$n = 3$	0	110	0	51	0	46	0	34	0	43	0	26	0	20	1	6	0	11	1	4
	0 %		0 %		0 %		0 %		0 %		0 %		0 %		17 %		0 %		25 %	
$n = 4$	0	62	0	39	0	22	1	36	4	15	4	13	2	9	2	5	4	8	0	0
	0 %		0 %		0 %		3 %		27 %		31 %		22 %		40 %		50 %		N/A	
$n = 5$	0	40	0	21	0	16	0	9	1	9	2	7	3	7	1	2	2	5	2	3
	0 %		0 %		0 %		0 %		11 %		29 %		43 %		50 %		40 %		67 %	

Supplementary Table 3. Characteristics of the jumping rates N_n on sample T4. For each range of drop radii, we provide the number of coalescences and jumps for $n = 3$, 4 and 5, denoting n as the number of drops involved in the coalescence.

Supplementary discussion

Supplementary Table 1 details the dimensions of the structures (pitch p, height h, top diameter l when applicable, cone angle β) along with the advancing and receding water contact angles θ_a and θ_r after the hydrophobic treatment. Apart from E0 (see paragraph 5), all nanocones from the H and the E families exhibit high contact angles and low hysteresis $\theta_a - \theta_r$, a manifestation of their superhydrophobicity. For extruded surfaces with a common pitch, similar advancing angles are observed, while the receding angles vary slightly. Conversely, surfaces H1 to H3 have an identical cone shape but different sizes – the higher they are, the larger θ_r. Increasing the height h decreases the area fraction of liquid on solid for a drop lying on the cone tops. A lower contact tends to increase the receding angle, which explains why E3 has the largest observed θ_r. Note finally that families H and E intersect, H3 and E2 being the same sample.

Similar to cones, the T family also exhibits high advancing contact angles, but the receding angles have much lower values. The hysteresis roughly triples when passing from the H/E families to the A/T families. This difference originates in the pinning of the contact line at the top edge of the truncated cone, as also seen with sample A, made of pillars.

During ESEM imaging, electrons can interact not only with water but also with the sample, which generates serious limitations[1-3] and contamination of the sample. Prolonged direct electron beam exposure of a superhydrophobic surface can lead to the carbonization of the irradiated area (amorphous carbon film formation)[4-5] or to the dissociation of the silanes coating our materials[6]. Supplementary Figure 1 shows such a degradation on sample H1. It reveals a darker square area, which was exposed for few minutes to a beam with an energy of 15 keV. The dark stains show condensation and evidence that water wets totally the surface, as demonstrated by the film-wise configuration. This morphology starkly contrasts with the behaviour expected on an antifogging surface where droplets exhibit large contact angles, even during condensation. Thus, the observed "hydrophilic" behaviour underlines the degradation of the hydrophobic coating. To avoid this predicament, one must use a low-energy electron beam (< 15 keV) and change the areas to which the beam is exposed during the whole condensation experiment. Radiation damage is another concern and it can hinder the ESEM imaging of condensation. The use of water vapor as surrounding gas can induce

inelastic e-beam scattering, which forms ionized or excited water molecules that decay into free radicals or ions[7]. The hydroxyl free radical ·OH is found to be the most aboundant[7-8]. Water radiolysis can damage the solid (change of wettability, for instance), as well as decrease the signal quality. To cope with this issue, a low energy electron beam is preferable since it will decrease the concentration of damaging species[7]. The beam-sample interaction can generate an accumulation of the net charge at the solid surface, a result of nonzero balance between incoming and outgoing electrons. The sample charge can either be positive or negative. If negative, the incoming primary electron beam can lose energy, hence causing a reduction in the penetration of the beam and an increase in SE emission. In extreme cases, the electric field at the sample surface is so high that the primary beam is actually deflected by the sample, which provides a deterioration of the image[9-10]. Besides, Stokes *et al.* evidenced a dynamic liquid charging during ESEM experiments[9], which can modify the wettability of the surface. We observed during our condensation experiments that changing the electrostatic potential (bias), that is, the potential imposed to the detector to collect electrons, can indeed induce dramatic changes in wettability (samples become hydrophilic). To prevent electrical charging, a low beam energy (< 15 keV) is required, as well as a low detector potential, even if the latter may decrease the quality of the images. The beam can also cause a significant heating and evaporation of water, especially when imaging a small area ($< 5 \times 5\ \mu m^2$).

ESEM images (figure 2 of the accompanying paper) suggest a robust Cassie state for droplets condensing on the nanocones of sample E4. In order to check whether this observation is universal, we complemented our study by ESEM observations on samples E2' and E3. Images of water condensing on both samples (Supplementary Figure 2a and 2b, respectively) confirm our conclusions: micrometric droplets all exhibit large contact angles, around 170°, a manifestation of their non-wetting Cassie states.

We complement the results in figure 3 (contact angles measured by ESEM imaging) by adding data obtained on materials E2' and E3 and compare them with those obtained on E4. Supplementary Figure 3 shows the advancing angle θ_a as a function of droplet radius r for the three samples. Remarkably, all data collapse on the same curve for radii smaller than 2 µm. For larger radii, data approach the values measured on millimetric drops and drawn in dotted

lines. These drops have a slightly smaller contact angles than that on E4, and we indeed expect that smaller angles reduce the discrepancies between micro and milli-measurements of angles, as discussed in the accompanying paper.

We complement our observations by ESEM imaging of drops condensing on truncated cones (Supplementary Figure 4a), either on T2 (top) or on T4 (bottom). All droplets exhibit large contact angles and their quasi-spherical shape indicates a Cassie state. This is further confirmed by plotting the advancing contact angle θ_a as a function of the droplet radius r (Supplementary Figure 4b). For both materials, θ_a increases with r and reaches 160° for radii as small as 2 µm, close or slightly larger than the value measured with millimetric drops (dotted lines), as in figure 3 for E4. Our model (eq. 1) predicts that water penetration z scales as hp/r. In order to prevent penetration, we need to minimize the product of h by p, that is, to miniaturize even more the texture design. This would imply structures smaller than 100 nm, that is, at a scale where long-range forces (such as van der Waals forces) influence the behavior of the liquid, and favor the so-called spinodal dewetting of water on these hydrophobic solids (as seen for instance in the simulations by Prakash *et al.*[11]). Such materials are beyond the current technology: with the pitch continuing to decrease, the electrostatic interference from neighboring features becomes more significant, being just one reason to explain the general challenges arising in profile controls, in addition to the stochastic nature of the etchant fluxes.

In the case of smaller cone angle β (very sharp needles), eq. (1) predicts that water penetrates the network of cones, as shown in Supplementary Figure 6b for $\beta = 1°$ (black line), 10° (red line) and 15° (blue line). The penetration of water is physically due to the fact that a small apex angle cannot oppose water penetration, since the liquid then is not stopped by a significant surface area of solid as it is at larger β. As further discussed, larger angles geometrically get invaded by water, which explains why only intermediate cone angles (as in our study) seem to be optimal for generating anti-fogging.

Antifogging behavior is observed on a dense array of sharp nanocones and we discuss here what happens on similar texture, yet with much higher apex angle. To that end, we achieved a compressed version of the cones of the extruded family, E0 (Supplementary Figure 7a), for

which the pitch is still p = 110 nm but the height only h = 101 nm, so that the apex angle is $\beta = 57° \pm 2°$. The sharp tip of the nanocone geometry texture enables the contact line to find a (unique) stable position, contrasting with nanopillars where the contact line can adopt different positions on the texture edges. Repellent materials are such that surface tension forces are oriented upwards to counter the pressure induced by liquid. The force exerted by the solid on the surface makes an angle $\pi - \theta_0 + \beta/2$ with the vertical, and it must be higher than $\pi/2$, hence yielding as a condition for superhydrophobicity: $\beta < \beta_c = 2\theta_0 - \pi$. At large β, the capillary force acts downwards so as to push water inside the texture and to induce a sticky Wenzel state. In our case, we have $\theta_0 \approx 120° \pm 2°$, which yields a critical apex angle $\beta_c = 60° \pm 4°$.

Our sample E0 has an apex angle β slightly smaller than β_c, and Supplementary Figure 7b confirms that water makes a markedly smaller advancing contact angle (*ca.* 130°) than on sharp cones. Even more clearly, hysteresis on E0 is greater than 100° (see Supplementary Table 1), showing unambiguously that water impregnates the texture (Wenzel state). In such conditions, one would expect for E0 a non-repellent character towards fog and Supplementary Figure 7c shows that indeed, after 25 min of condensation, the surface is covered with large and deformed (as noted by the yellow dotted circle) droplets.

We collected our results and models in a phase diagram of jumping, based on the texture parameters h, p, l and $\Sigma = 1/\beta$. Three of those parameters can be tuned independently, so that the phase diagram is three-dimensional, which complicates its readability. We simplify the diagrams by considering successively sharp cones ($l \approx 0$) and truncated cones.

In the first case, antifogging is possible provided the cone dimensions are within two limits fixed by the apex angle. If β is too large, the material is no longer Cassie stable and drops wets totally the surface, that is, when β is larger than $60° \pm 4°$ ($\Sigma < \sim 1$). Another limit emerges when the cones are very sharp (small β, large Σ). Then, our model predicts that drops sink deep in the structures (Supplementary equation 1 and Supplementary Figure 6b), which impedes their motion and prevents jumping. As a criterion of immobilization, we choose that the water penetration depth z becomes at least 10% of the drop radius r for $r < 1.5$ µm, which yields a critical apex angle of 4° ($\Sigma \approx 7$). Finally, these two limits are expressed through the relationship $h = p/2 \tan(\beta/2)$, with $\beta \approx 4°$ and $\beta \approx 60°$, drawn with solid lines in the phase

diagram based on the cone height h and pitch p, and constituting the Supplementary Figure 8a. Green and red colors respectively distinguish jumping from non-jumping, and we add our data points for the E and H families – all found to be in the jumping region (green data), except one (red data), indeed found to be at the boundary between jumping and non-jumping.

In the second case, the top diameter l is non null. Firstly, a geometric limit for truncated cones is imposed on l, that is, $l < p$. Secondly, for a given material, we assume that the advancing angle θ_a has to be large enough to enable jumping, and we choose a value of 150° (often used as the superhydrophobic condition) as a criterion of jump. Using eq. (2), this yields a critical solid fraction $\varphi_S = \pi l^2/2\sqrt{3}p^2$, which provides a relation between p and l. This allows us to build a second phase diagram for truncated cones (Supplementary Figure 8b), where the dotted line is the equation $l = p$ and the solid line the equation based on the critical solid fraction and for which θ_a is equal to 150°. We have not considered the cone height h in our analysis, but it is fixed by the two limits given in figure S8a: $h = (l - p)/2 \tan(\beta/2)$, where β is 4° or 60°. The four data for which jumps are observed indeed lie in the green domain of jumping.

We can compare the breath figures for three families with contrasting antifogging abilities: pillars, homothetic and truncated cones. Firstly, we focus on pillars and the homothetic family, of low and high antifogging efficiency, respectively. In Supplementary Figure 9, we report the breath figures captured by optical microscopy for sample A and samples H1-H3 as a function of time. All samples are brought to a temperature of 4°C, and water condenses from the atmosphere. As expected, the previously observed behaviours are reflected in the breath figures: pillars are largely covered by water after 30 min, indicating yet another time their poor antifogging efficiency, as already shown by the low contact angles (figures 2 and 3) and the negligible jumping rate N_g (figure 4b). Contrastingly, no distinct differences can be noted between the three homothetic samples H. In all cases, the drop size does not diverge, but rather reaches a maximum (on the order of 30 μm in radius), a consequence of the high jumping rate that constantly evacuates water condensates from the materials.

Comparing to the observations on truncated cones (family T; Supplementary Figure 10), drops reach a size intermediate between that on A and H. However, the drop size on T1 (for

which the global jumping rate is ~7%, see figure 4b) remains modest, slightly larger than on H, but significantly smaller than on T2-T4 and A. As developed in the accompanying paper (figure 4c), we attribute this behavior to the high jumping rate of multiple coalescences, in particular at large drop radius (r> 15 μm), which explains why droplets are eventually comparable to that in Supplementary Figure 9. Hence breath figures appear to be a qualitative tool for discriminating the antifogging efficiency of materials.

We measured the initial density of nucleation on all samples. Here again, families H and E behave in a similar fashion: the density of nuclei per unit area is roughly constant on all samples, with a value of 1200 mm^{-2} (which implies an average distance between nuclei of ~30 μm). However, the density of nucleation changes when tops are present on the texture (either for pillars, or for truncated cones), with values of 2300 mm^{-2} (truncated cones, sample T1) and to 5600 mm^{-2} (pillars). Hence the presence of tops favors nucleation and increases its density by respective factor of 2 and 5, when passing from cones to truncated cones and to pillars. The link between the nucleation map and the antifogging properties is not obvious: most drops depart with a radius much smaller than 30 μm, due to fluctuations in the distance between nucleation sites. However, it is interesting to note that the maximum size of drops departing on cones is roughly 35 μm, that is, comparable to the mean distance between nucleation sites.

The stationary character of our curves showing the jumping rate as a function of time suggests to look at the nucleation "persistence": we measured the probability of observing a droplet renucleating on a site where a droplet had previously nucleated before taking off. We concentrated on the initial set of nuclei, which defines the "hydrophilic map" of a sample. We consider a population of 150 initial nuclei on sample H1 and report the number of new nuclei appearing at their location, after drops depart. We present in Supplementary Figure 11 the histogram of renucleation: the probability of having at least one renucleation is as high as 96% and these sites are found to be extremely active, since 35% of them will give more than 10 renucleations. We checked that the renucleation probability is comparable on H1 and E4 (with respective values of 94% and 96%). Hence, the initial set of nucleation sites remains "robust" and well-defined throughout the condensation – contributing to the explanation for our experimental reproducibility.

It is worth comparing these numbers with the probability of renucleation on a site that did not originally contain a nucleus. We could observe about 100 droplets on samples H1 and E4 that appeared suddenly at a given “virgin” place of the sample, the result of a coalescence that propelled drops along the sample rather than perpendicular to it. We find respective renucleation probabilities of 7% and 6%, respectively. These low values confirm that nucleation is privileged on active sites, which makes the process repetitive, reproducible and stationary.

We now focus on the absolute numbers of events deduced from breath figures. We report in Supplementary Figure 12 the number of drops on conical and pillar structures, showing respectively anti-fogging and regular condensation behaviors. On sample H1, the absolute number of drops first rises (nucleation phase), then decreases (start of the jumps) and eventually plateaus (repetitive sequences of nucleation/growth/depart, as discussed above). The stationary regime is reached after typically 10-15 minutes (300 drops approximately), a small time compared to the total duration of observation (45 minutes). On sample A (nanopillars) where very few jumps are observed, the drop number continues to decrease – which corresponds to coalescences without departure.

We also provide in Supplementary Figure 13 the absolute numbers of coalescences and jumps as a function of time for samples H1, H3/E2 and E4. Data globally match the trends observed in Supplementary Figure 12 (rise, decrease and plateau). However, we also observe strong fluctuations of both numbers of events, due to the intrinsic nature of the phenomenon: for instance, after one large drop leaves, many tiny drops appear, coalesce and jump, which generates a puff of events. However, the three samples show a strong correlation between the two kinds of events (regardless the amplitude of the fluctuations), which justifies our choice of the global jumping rate N_g as a metric for anti-fogging – a way to gather and compare our results, but also to provide a practically useful quantity, namely the rate of departure.

Interestingly, the number of events is slightly larger on H1, owing to a slightly larger density of nucleations (1000 mm^{-2} compared to the two others, 800 mm^{-2} and 900 mm^{-2}, respectively). However, if we compare the percentage of jumps (figure S14, next section), they are all similar, which points out the universality of the reported phenomena for sharp nanocones.

We now complement the experiments of the accompanying paper by reporting the global jumping rate N_g as a function of time t for the homothetic and extruded families of nanocones (Supplementary Figure 14a). Each data point is obtained after averaging the proportion of jumps over one minute (about 100 jumps). For all materials, N_g is found to be roughly constant and around 90%, which reveals a good stability of the antifogging properties and confirms the generality of the observations reported in figure 4. More precisely, the average values of N_g over 30 minutes are 90%, 84% and 88% for samples H1 to H3, and 89%, 88%, 91% and 89% for samples E1 to E4. Similarly, plotting the jumping rate N_2 for symmetric coalescences as a function of the mean radius $<r>$ (Supplementary Figure 14b) does not enable us to differentiate between the homothetic and the extruded families, which speaks to the universal antifogging behaviour of nanocones.

Another (less sensitive) way to probe the antifogging efficiency of a material is to determine the area fraction ϕ covered by water, which defines the optical properties of a transparent surface: the larger ϕ, the larger the area where light will be deviated by water. For the three homothetic samples H1-H3, we measure ϕ as a function of time t (Supplementary Figure 15a). At the beginning, ϕ is the same on all samples and it increases with time, albeit a bit higher for H2 because of a larger nucleation density. After growing, ϕ stabilizes around a constant value that hardly differs between the three surfaces: ϕ = 30, 31 and 36 % for H1, H2 and H3, respectively. The non-monotonic evolution of ϕ with the pitch p does not enable us to determine a possible influence of the texture size on dew repellency. For the extruded family, the area fraction ϕ covered by water is found in Supplementary Figure 15b to be similar for the four samples: $\phi = 34 \pm 4$ %, a value close to that on the homothetic family (figure S15a).

The anti-fogging performance can also be measured over a longer period. In Supplementary Figure 16, we test E4 for a duration ~5 times longer compared to previous experiments. In Supplementary Figures 16a and 16b, we report the evolution of the global jumping rate N_g (ratio between numbers of jumps and coalescences) and of the area fraction ϕ covered by water drops. We find that the stationary regime evidenced in Supplementary Figures 14 and 15 remains valid throughout the experiment. For instance, the plateau value of N_g (averaged over time) is 92%, instead of 89% in Supplementary Figure 14. In addition, these measurements were done 18 months after that reported in figure 4, Supplementary Figures 14

and 15, during which the sample was simply stored in a closed box. Data can be fully superimposed, showing that the anti-fogging efficiency can be maintained over long durations.

We now examine the influence of supersaturation on jumping efficiency. The nucleation density is observed to decrease with lower supersaturation, ranging from 730 to 920 mm^{-2} and from 1000 to 5400 mm^{-2} for S between 1.1 and 1.6, on E4 and A (nanopillars) respectively. We also plot in Supplementary Figure 17 the global jumping rate N_g as a function of time for different supersaturations and the two samples E4 and A. On E4 (Supplementary Figure 17a), N_g exhibits a high value over time for the two values of S. A slightly larger mean value of N_g is observed for the lowest value of S, being around 96% against 89% for $S = 1.6$. On material A (Supplementary Figure 17b), the influence of S on N_g remains also modest; despite lower supersaturations, the percentage of jumps is still very low, which empahsizes the inability of material A to repel dew at all temperatures.

Contact angles on truncated cones are large enough to potentially yield anti-dew behavior. However this behavior is less than that of H/E families. This is likely due to the pinning on the pillar tops, a property evidenced by the significant angle hysteresis on the T family, which reduces the mobility of the drops. Figure 4c shows that an original antifogging mechanism reconciliates these contradictory observations. We complement this figure by collecting in Supplementary Table 2 all the numbers of jumps and coalescences across all radii. At large n (the number of drops involved in a coalescence), there are few events, typically 10, especially for "large" drops, which can induce significant fluctuations in the data.

To compare with the observations made on T1 in figure 4c (also in Supplementary Figure 18a), we provide in Supplementary Figure 18b the jumping rate for the sample T4, that is, a much more truncated cone. Our mechanism for droplet ejection remains valid (multiple coalescences are responsible for water evacuation), and the lower performance of this sample is explained by a shift of the jumping rate towards larger droplet radii. We also provide a table for this new set of data (Supplementary Table 3).

The contact angles measured in figure 3 can be measured for larger drop radii, as shown in Supplementary Figure 19a for materials A and E4. The saturation observed in figure 3 at "large" radii is confirmed in these experiments where *r* is augmented by a factor of two and three, respectively. The values at the plateau (dotted lines) are $\theta_a = 141° \pm 6°$ for A and $\theta_a = 169° \pm 6°$ for E4, in close agreement with values extracted in figure 3.

In the same spirit, we can also discuss the jumping rate after a binary coalescence for drops much larger than previously discussed in figure 4 (Supplementary Figure 19b). On nanocones (blue data), there is a cut-off radius above which we do not observe any drops: the antifogging efficiency of these materials prevents the formation of drops larger than ~25 µm – a distance that can be considered as another metric of antifogging. On nanopillars (sample A, red data), drops can grow without departing, which allows us to follow their behavior in the range of 10-100 µm. It is found that the jumping rate remains zero whatever the drop size so that this exploration of larger drop radii does not modify the conclusions expressed in the accompanying paper.

However, it was reported in the literature that large enough droplets can depart from nanoneedles (nanopillars with a high aspect ratio). We analyzed the movies of ref. 12, where the global jumping rate of droplets departing nanoneedles is 36%. We report in Supplementary Figure 20 the departing rate after a binary coalescence established from the observation of 117 events. Contrasting with our observations on short nanopillars, droplets can depart if their radius is large enough (above 5 µm, significantly larger than the 1.5 µm on nanocones), but the small number of observed coalescences makes it difficult to establish precise statistics for each size, as done in the figure 4 of the accompanying paper where we analyzed thousands of coalescences. The increase of jumping might agree with the observation that the contact angle increases with the drop size – but the absence of model for jumping as a function of contact angle makes it difficult to go beyond this qualitative observation. Long needles may also be flexible, which can promote the expulsion of large enough droplets from the core of the structures and thus enhance their mobility.

In order to further test the versatility of the nanocone design towards dew-repellency, we tested material E2', which belongs to the extruded family with similar dimensions as E2, but possessing a different shape: as seen in Supplementary Figure 21a, cones have a non-constant apex angle β. The sidewalls are convex instead of straight, and β decreases continuously from

the bottom to the top of the cones. This particular geometry is worth testing in order to fully assess the fog repellency of the conical nanotexture. As such, we compare in Supplementary Figure 21b the antifogging repellency of both samples E2 and E2' through the jumping rate N_2 as a function of $<r>$, the mean radius at coalescence for binary, symmetric coalescences. Similar to figures 4c and 4d, results collapse on a single curve and we find for E2' a critical radius $r_c = 1.5 \pm 0.3$ μm, very close to that measured for E2, $r_c = 1.6 \pm 0.3$ μm. This further observation then strengthens the universality of dew-repellency on sharp, dense nanocones.

Supplementary Movie

Breath figures on samples E4, T1 and T4. Supersaturation is maintained at a constant value $S = 1.6 \pm 0.2$. The real duration of the experiment is 30 min.

Supplementary References

1. Egerton, R.F., Li, P. & Malac, M. Radiation damage in the TEM and SEM. *Micron* **35**, 399-409 (2004).
2. Stokes, D. Principles and practice of variable pressure/environmental scanning electron microscopy (VP-ESEM). *John Wiley & Sons* (2008).
3. Barkay, Z. Nanodroplets. *Springer*, pp. 51-72 (2013).
4. Aronov, D., Rosenman, G. & Barkay, Z. Wettability study of modified silicon dioxide surface using environmental scanning electron microscopy. *J. Appl. Phys.* **101**, 084901 (2007).
5. Aronov, D., Molotskii, M. & Rosenman, G. Electron-induced wettability modification. *Phys. Rev. B* **76**, 035437 (2007).
6. Bozso, F. & Avouris, P. Thermal and electron-beam-induced reaction of disilane on Si(100)-(2x1). *Phys. Rev. B* **38**, 3943-3947 (1988).
7. Royall, P., Thiel, B.L. & Donald, A. Radiation damage of water in environmental scanning electron microscopy. *J. of Microscopy* **204**, 185-195 (2001).
8. Donald, A. & Kitching, S. Beam damage of polypropylene in the environmental scanning electron microscope: a FTIR study. *J. Microscopy* **190,** 357-365 (1998).
9. Stokes, D.J., Thiel, B.L. & Donald, A. Dynamic secondary electron contrast effects in liquid systems studied by environmental scanning electron microscopy. *Scanning* **22,** 357-365 (2000).
10. Goldstein, J.I., Newbury, D.E., Michael J.R., Ritchie N.W.M., Scott J.H.J. & Joy D.C. Scanning electron microscopy and X-ray microanalysis. *Springer* (2017).
11. Prakash, S., Xi, E. & Patel, A.J. Spontaneous recovery of superhydrophobicity on nanotextured surfaces. *Proc. Natl. Acad. Sci.* **113**, 5508-5513 (2016).
12. Enright, R., Miljkovic, N., Al-Obeidi, A., Thompson, C.V. & Wang, E.N. Condensation on superhydrophobic surfaces: the role of local energy barriers and structure length scale. *Langmuir* **28**, 14424-14432 (2012).
