# Supplementary material for: Unique and universal dew-repellency of nanocones
Source: Nat Commun. 2021 Jun 8;12:3458. doi: 10.1038/s41467-021-23708-6 (PMC8187394; doi:10.1038/s41467-021-23708-6)
Supplement: Supplementary file 3 — Description of Additional Supplementary Files [file 41467_2021_23708_MOESM3_ESM.pdf]

Description of Additional Supplementary Files

Supplementary Movie 1

Breath figures on samples E4, T1 and T4. Supersaturation S is maintained at a constant value 1.6 ± 0.2. The real duration of the experiment is 30 min.
